# Robotic vs. 3D laparoscopic resection for rectal cancer: a single-center retrospective study of short-term outcomes and functional recovery

**DOI:** 10.3389/fsurg.2025.1630237

**Published:** 2025-07-31

**Authors:** Dunbo Liu, Qianshi Zhang, Fangliang Guo, Zhiwei Sun, Shuangyi Ren

**Affiliations:** Department of General Surgery, The Second Hospital of Dalian Medical University, Dalian, China

**Keywords:** rectal cancer, robotic surgery, tumor resection, evaluation of efficacy, retrospective analysis

## Abstract

**Background:**

Within the evolving landscape of precision medicine, robot-assisted surgery has emerged as a valuable tool in rectal cancer management. However, comprehensive evidence regarding its safety and clinical efficacy remains limited.

**Methods:**

This retrospective study analyzed 235 patients with middle and low rectal cancer who underwent surgical resection from January 2020 to March 2023. Comprehensive perioperative data, including baseline characteristics, intraoperative parameters, and postoperative outcomes, were systematically collected and analyzed. The International Prostate Symptoms Score, International Erectile Function Index, and Female Sexual Function Index were utilized to assess postoperative organ function recovery. Multivariable logistic regression analysis was performed to identify predictors of functional recovery and postoperative complications. All patients were prospectively followed for up to 3 years, with survival outcomes evaluated using Kaplan–Meier analysis.

**Results:**

Robotic surgery demonstrated significantly longer operative duration compared to 3D laparoscopic surgery (162.0 ± 44.0 vs. 149.0 ± 41.0 min, *p* < 0.05), yet resulted in significantly reduced intraoperative blood loss (51.0 ± 34.0 vs. 63.0 ± 43.5 ml, *p* = 0.010), albeit with substantially higher procedural costs (93,808 ± 1,334 vs. 71,863 ± 1,220 yuan, *p* < 0.05). Notably, the robotic approach yielded superior lymph node retrieval, facilitated earlier detection of pathological stages, promoted enhanced recovery of urogenital function, and was associated with a reduced incidence of severe postoperative complications. Multivariable analysis revealed that robotic surgery was an independent predictor of improved urinary function recovery at 3 months (OR = 3.45, 95% CI: 1.82–6.54, *p* < 0.001) and enhanced sexual function recovery at 6 months (male: OR = 2.89, 95% CI: 1.41–5.93, *p* = 0.004; female: OR = 3.12, 95% CI: 1.23–7.89, *p* = 0.017). Both surgical approaches demonstrated comparable baseline characteristics and long-term survival outcomes (*p* > 0.05).

**Conclusion:**

Robotic surgical systems demonstrate comparable safety and efficacy to traditional laparoscopy for rectal cancer resection, while offering distinct advantages including reduced intraoperative bleeding, improved pathological staging accuracy, and accelerated postoperative recovery of urinary and reproductive function. Both procedures exhibit similar short-term efficacy and safety profiles, with no significant difference in long-term survival rates.

## Introduction

Rectal cancer represents the third most prevalent malignancy globally, with a concerning trend toward younger age of onset. Over recent decades, the mortality rate of rectal cancer has exhibited a sustained upward trajectory, establishing it as the fifth leading cause of cancer-related death and presenting a serious challenge to global public health (1, 2). Throughout the past several decades, treatment approaches for rectal cancer have undergone significant diversification. The advent of novel therapeutic modalities, including neoadjuvant chemoradiotherapy and targeted therapy, has substantially enhanced both the postoperative quality of life and prognosis for rectal cancer patients (3, 4). Despite these advances, surgery remains the cornerstone of rectal cancer treatment. Total mesorectal excision (TME) has been established as the gold standard for curative surgical treatment of rectal cancer (5).

In comparison to traditional open surgery, laparoscopic TME confers multiple advantages, including reduced intraoperative bleeding and decreased risk of wound infection, leading to its widespread adoption in contemporary clinical practice (6, 7). Nevertheless, particularly in patients with mid-to-low rectal cancer, the preservation of pelvic autonomic nerves remains a formidable technical challenge during laparoscopic surgery, despite its minimally invasive advantages over open surgery (8).

In response to these limitations, robotic surgical systems have emerged as an innovative solution. By leveraging distinctive technological advantages, including high-definition 3D visualization and articulating instruments with multiple degrees of freedom, these systems facilitate meticulous dissection within the confined pelvic environment (9, 10). However, current comparative studies between robotic and 3D laparoscopic treatment of rectal cancer have predominantly focused on short-term outcomes and survival analysis, with limited comprehensive assessments of urologic and sexual function (11–13). Currently, large-sample studies remain insufficient to comprehensively elucidate the safety and functional preservation capabilities of robotic surgical systems in the treatment of low rectal cancer.

Therefore, this study aims to comprehensively evaluate the safety and feasibility of robot-assisted proctectomy (RAP) vs. 3D laparoscopic-assisted proctectomy (3D LAP) in the treatment of middle and low rectal cancer by systematically comparing short-term clinical efficacy, recovery of urinary and sexual function, and long-term prognosis after surgery. Our findings aim to provide evidence-based guidance for surgical decision-making in rectal cancer treatment.

## Methods

### Patients

This retrospective cohort analysis was approved by the institutional review committee of the Second Affiliated Hospital of Dalian Medical University. Before enrollment, all participants underwent comprehensive screening, including upper endoscopy and CT scans of the abdomen and chest. Written informed consent was obtained from all participants after ensuring their complete understanding of the study protocol. All procedures were conducted in strict accordance with the ethical guidelines established by the hospital ethics committee.

### Surgical procedures

Both types of surgery were performed by the same team of experienced minimally invasive surgery specialists. Robotic surgery was performed using the fourth-generation da Vinci Xi surgical robot system (Intuitive Surgical Inc., Sunnyvale, CA, USA), while the laparoscopic approach utilized the Storz 3D system (Germany). Both surgical approaches were conducted in accordance with the consensus of Chinese experts on robotic colorectal cancer surgery (2015 and 2020 editions) and the guidelines for laparoscopic radical resection of colorectal cancer (2018 edition) (14, 15).

### Statistical analysis

SPSS 26.0 statistical software was used for data analysis. Data conforming to normal distribution are presented as mean ± standard deviation (X ± SD). Survival analysis was performed using the Kaplan–Meier method. Fisher's exact test or chi-square test was used to compare categorical variables, which are expressed as numbers with percentages. Multivariable logistic regression analysis was conducted to identify independent predictors of functional recovery and postoperative complications. Variables with *p* < 0.1 in univariate analysis were included in the multivariable model. Odds ratios (OR) with 95% confidence intervals (CI) were calculated. A *p*-value less than 0.05 was considered statistically significant.

## Results

### Clinical baseline characteristics and short-term surgical outcomes

A total of 235 patients met the inclusion criteria, comprising 110 patients in the 3D LAP group and 125 patients in the RAP group. Statistical analysis of preoperative clinical baseline data revealed no significant differences between the groups regarding gender, age, body mass index, history of abdominal surgery, or ASA grade (Table 1, *P* > 0.05). As anticipated, the higher initial investment and operational costs in the RAP group demonstrated a statistically significant difference (*P* < 0.001).

**Table 1:** Comparison of baseline data and short-term outcomes.

Features	RAP group (*n* = 125)	3D LAP group (*n* = 110)	*P* value
Gender			0.342
Male	79 (63.2%)	76 (69.1%)	
Female	46 (36.8%)	34 (30.9%)	
Age (years)	63.0 ± 10.0	64.0 ± 9.0	0.625
Body mass index (kg/m^2^)	24.5 ± 2.7	24.0 ± 3.2	0.060
Serum albumin level (g/L)	39.5 ± 2.1	39.5 ± 1.4	0.669
History of abdominal surgery (%)			0.357
Yes	20 (16.0%)	13 (11.8%)	
No	105 (84.0%)	97 (88.2%)	
Hospitalization expenses (¥)	93,808 ± 1,334	71,863 ± 1,220	<0.001
ASA classification (%)			0.992
Ⅰ	69 (55.2%)	60 (54.5%)	
Ⅱ	49 (39.2%)	44 (40.0%)	
Ⅲ	7 (5.6%)	6 (5.5%)	
Operative time (min)	162.0 ± 44.0	149.0 ± 41.0	0.034
Operative blood loss (ml)	51.0 ± 34.0	63.0 ± 43.5	0.010
First exhaust time (d)	2 (1, 3)	3 (2, 3)	<0.001
Defecation time (d)	3 (1, 6)	3 (1, 7)	0.562
Unplanned readmission within 30 days (%)	5 (4.0%)	5 (4.5%)	0.825
Postoperative length of stay (d)	7.5 ± 1.2	7.0 ± 1.3	0.123
Median time to remove urinary catheter (d)	3.3 ± 1.7	3.3 ± 1.5	0.894

Analysis of intraoperative and postoperative parameters revealed that operative time in the RAP group was significantly prolonged compared to the 3D LAP group (*P* < 0.05). Remarkably, despite the extended operative duration, the RAP group demonstrated significantly reduced blood loss compared to the 3D LAP group (*P* < 0.05).

With respect to postoperative recovery, the first exhaust time in the RAP group was significantly shorter than that observed in the 3D LAP group, representing a statistically significant difference. No statistically significant differences were observed in defecation time, unplanned readmission rates, postoperative hospital stay, or time to urinary catheter removal (*P* > 0.05).

### Postoperative pathological findings

Histopathological analysis revealed no significant differences in the number of retrieved lymph nodes, degree of tumor differentiation, or distal margin distance between the groups (Table 2, *P* > 0.05). Of particular note, significant statistical differences were observed in tumor staging and tumor diameter between the groups (*P* < 0.05).

**Table 2:** Comparison of postoperative pathological outcomes between the two groups.

Features	RAP group (*n* = 125)	3D LAP group (*n* = 110)	*P* value
Numbers of retrieved lymph nodes	18.7 ± 8.2	20.3 ± 9.2	0.060
Tumor diameter (cm)	4.0 ± 1.4	4.5 ± 1.8	0.015
Distal resection margin (cm)	2.0 ± 1.0	2.2 ± 1.0	0.070
Tumor differentiation (%)			0.206
Low degree	7 (5.6%)	11 (10.0%)	
Middle and high degree	118 (94.4%)	99 (90.0%)	
Tumor staging of AJCC (%)			0.030
Ⅰ	37 (29.6%)	21 (19.1%)	
Ⅱ	40 (32.0%)	53 (48.2%)	
Ⅲ	48 (38.4%)	36 (32.7%)	

### Postoperative complications

Perioperative complications represent a crucial metric for evaluating short-term efficacy. Table 3 presents a comprehensive analysis of postoperative complications. In this study, a total of 58 complications were documented across both groups (35 cases in the RAP group, 23 cases in the 3D LAP group), although this difference did not reach statistical significance (*P* > 0.05).

**Table 3:** Complications in RAP and 3D LAP groups.

Complications	RAP group (*n* = 125)	3D LAP group (*n* = 110)	*P* value
Total number of cases (%)	35 (28.0%)	23 (20.9%)	0.150
Heart failure (%)	1 (0.8%)	1 (0.9%)	
Pneumonia (%)	7 (5.6%)	1 (0.9%)	
Venous thrombosis (%)	4 (3.2%)	4 (3.6%)	
Urinary tract infection (%)	5 (4.0%)	4 (3.6%)	
Wound infection (%)	4 (3.2%)	1 (0.9%)	
Anastomotic leakage (%)	6 (4.8%)	4 (3.6%)	
Intra-abdominal infections (%)	3 (2.4%)	1 (0.9%)	
Anastomotic bleeding (%)	2 (1.6%)	2 (1.8%)	
Chylous leakage (%)	2 (1.6%)	0 (0.0%)	
Intestinal obstruction (%)	1 (0.8%)	1 (0.9%)	

### Multivariable logistic regression analysis

Multivariable logistic regression analysis revealed that robotic surgery was an independent predictor of improved functional recovery. After adjusting for age, BMI, tumor stage, operative time, and blood loss, patients undergoing RAP had significantly higher odds of achieving better urinary function recovery at 3 months (OR = 3.45, 95% CI: 1.82–6.54, *p* < 0.001), male sexual function recovery at 6 months (OR = 2.89, 95% CI: 1.41–5.93, *p* = 0.004), and female sexual function recovery at 6 months (OR = 3.12, 95% CI: 1.23–7.89, *p* = 0.017) compared to those undergoing 3D LAP (Table 4). Younger age was also associated with better urinary function recovery (OR = 0.78 per 10-year increase, *p* = 0.041).

**Table 4:** Multivariable logistic regression analysis for predictors of functional recovery.

Variables	Urinary function recovery at 3 months		Male sexual function recovery at 6 months		Female sexual function recovery at 6 months	
Category	OR (95% CI)	*P* value	OR (95% CI)	*P* value	OR (95% CI)	*P* value
Surgical approach (RAP vs. 3D LAP)	3.45 (1.82–6.54)	<0.001	2.89 (1.41–5.93)	0.004	3.12 (1.23–7.89)	0.017
Age (per 10 years)	0.78 (0.61–0.99)	0.041	0.82 (0.65–1.04)	0.102	0.85 (0.58–1.24)	0.394
BMI (per unit)	1.02 (0.94–1.11)	0.623	0.98 (0.89–1.08)	0.682	1.05 (0.91–1.21)	0.507
Tumor stage (III vs. I–II)	0.67 (0.42–1.07)	0.093	0.72 (0.38–1.36)	0.311	0.69 (0.31–1.54)	0.365
Operative time (per 30 min)	0.91 (0.74–1.12)	0.371	0.95 (0.76–1.19)	0.654	0.88 (0.64–1.21)	0.433
Blood loss (per 50 ml)	0.86 (0.68–1.09)	0.212	0.92 (0.71–1.19)	0.527	0.94 (0.67–1.32)	0.721

### Postoperative functional recovery assessment (urinary function)

**Figure 1:**
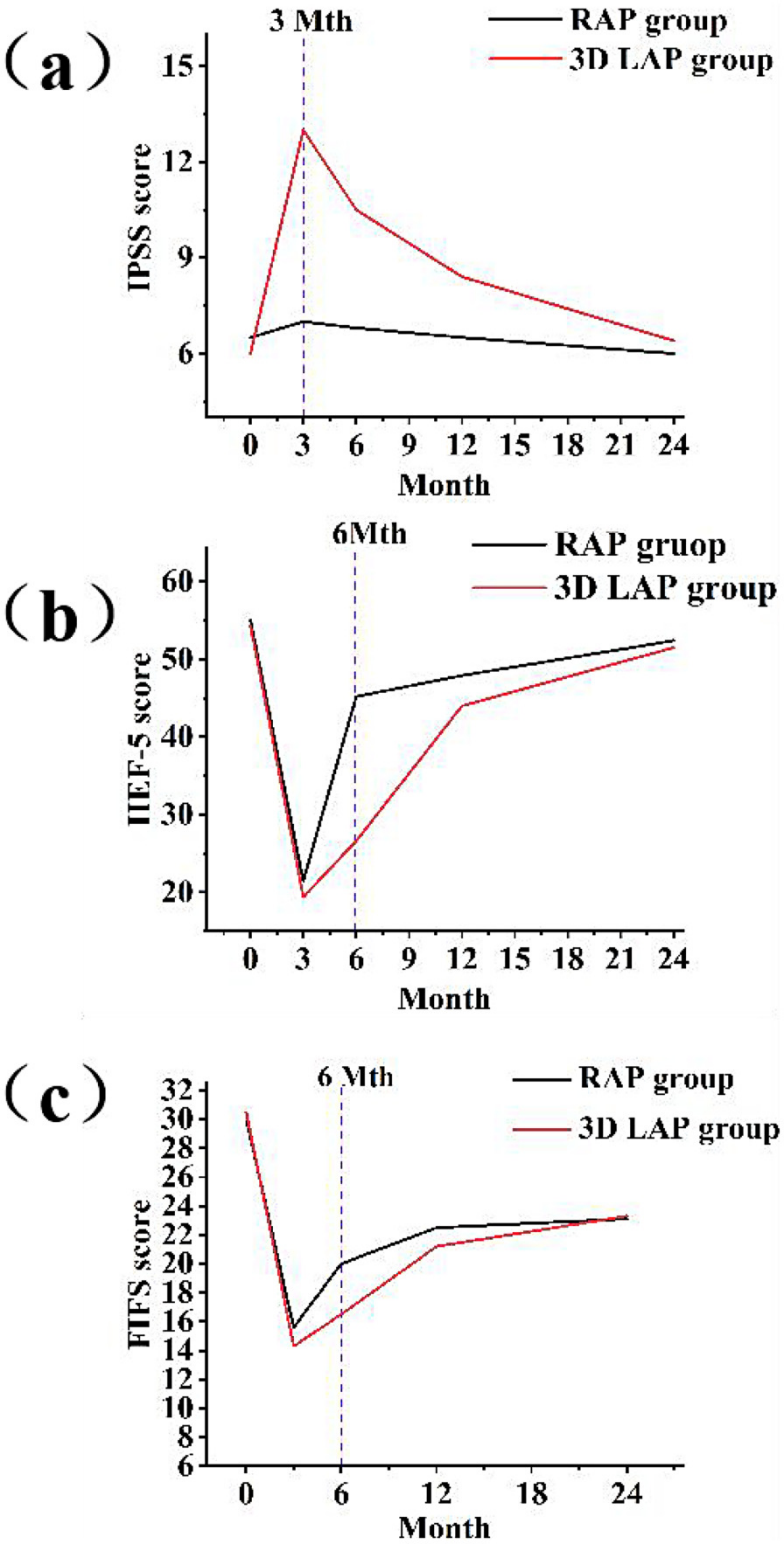
Postoperative functional recovery assessment using international prostate symptoms score **(a)**, international erectile function Index **(b)**, and female sexual function Index **(c)** three line graphs showing functional recovery over 24 months post-surgery: **(a)** IPSS scores comparing RAP (blue line) vs. 3D LAP (red line) groups, showing better urinary function recovery in RAP group **(b)** IIEF-5 scores for male patients, demonstrating superior erectile function recovery in RAP group. **(c)** FSFI scores for female patients, illustrating enhanced sexual function recovery in RAP group All graphs include error bars representing standard deviation and asterisks (*) indicating statistically significant differences (*p* < 0.05).

Given the intimate anatomical relationship between the pelvic autonomic nerves and the surgical field of total mesorectal excision (TME), there is an inherent risk of postoperative genitourinary dysfunction (16, 17). Among the 235 patients in this study, 210 successfully completed follow-up assessments of urinary function (110 cases in the RAP group, 100 cases in the 3D LAP group). At three months post-surgery, the IPSS score in the 3D LAP group was significantly elevated compared to preoperative baseline levels, demonstrating a statistically significant difference compared to the RAP group (*P* < 0.05). With progressive recovery over time, the total IPSS score in the 3D LAP group showed further improvement by six months compared to three months, yet remained higher than in the RAP group, though this difference lacked statistical significance. At the twelve-month postoperative assessment, urinary function in the RAP group had essentially returned to preoperative levels, with 95% of patients achieving complete functional recovery by 24 months (Table 5; Figure 1a).

**Table 5:** Evaluation of urinary function between RAP and 3D LAP groups.

Total IPSS	Preoperative scoring	Postoperative scoring (3 month)	Postoperative scoring (6 month)	Postoperative scoring (12 month)	Postoperative scoring (24 month)
RAP group (*n* = 110)	6.5 ± 1.0	7.0 ± 2.0	6.8 ± 0.8	6.5 ± 1.6	6.0 ± 2.0
3D LAP group (*n* = 100)	6.0 ± 0.8	13.0 ± 1.5	10.5 ± 0.5	8.4 ± 0.6	6.4 ± 1.5
*P* value	0.065	<0.001	0.242	0.655	0.195

### Postoperative functional recovery assessment (male sexual function)

Due to varying levels of patient cooperation, only 106 male patients from both groups participated in the erectile function evaluation questionnaire (Table 6) (18). The six-month postoperative evaluation demonstrated a statistically significant difference between the RAP and 3D LAP groups (Figure 1b).

**Table 6:** Evaluation of erectile function between RAP and 3D LAP groups.

IIEF-5 questionnaire score	Preoperative scoring	Postoperative scoring (3 month)	Postoperative scoring (6 month)	Postoperative scoring (12 month)	Postoperative scoring (24 month)
RAP group (*n* = 60)	55.0 ± 5.5	21.5 ± 4.1	45.2 ± 4.4	47.9 ± 4.8	52.4 ± 4.3
3D LAP group (*n* = 46)	54.3 ± 4.2	19.4 ± 4.5	26.6 ± 4.7	44.0 ± 4.1	51.5 ± 4.1
*P* value	0.611	0.103	<0.001	0.195	0.199

### Postoperative functional recovery assessment (female sexual function)

During the postoperative follow-up period, a total of 55 female patients underwent assessment of sexual function using the FSFI score (19) (Table 7). Preoperative sexual function assessment revealed no significant difference between the two groups (*P* > 0.05). At three months following surgery, although the FSFI score in the RAP group showed improvement compared to the 3D LAP group, this difference did not achieve statistical significance (Figure 1c). Notably, by six months post-surgery, FSFI scores continued to improve in the RAP group, resulting in a statistically significant difference between the groups, indicating that the robotic group demonstrated a more favorable trajectory in sexual function recovery.

**Table 7:** Evaluation of female sexual function between RAP and 3D LAP groups.

FSFI score	Preoperative scoring	Postoperative scoring (3 month)	Postoperative scoring (6 month)	Postoperative scoring (12 month)	Postoperative scoring (24 month)
RAP group (*n* = 30)	30.0 ± 5.5	15.6 ± 4.1	20.0 ± 4.4	22.5 ± 4.8	23.1 ± 3.7
3D LAP group (*n* = 25)	30.5 ± 4.0	14.3 ± 4.5	16.5 ± 1.7	21.2 ± 4.1	23.3 ± 4.1
*P* value	0.611	0.103	<0.001	0.395	0.199

### Survival analysis

**Figure 2:**
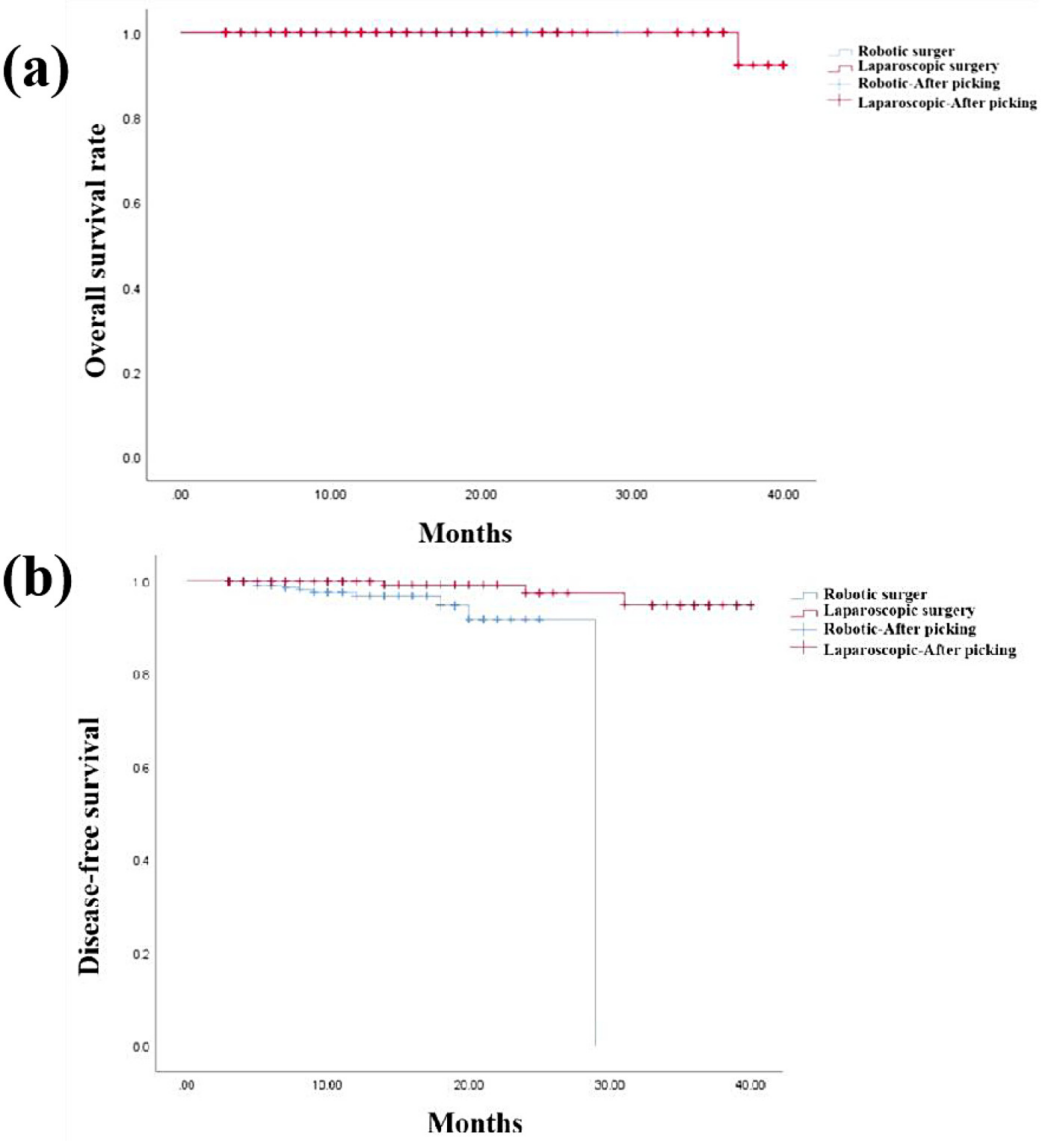
Overall survival rate **(a)** and disease-free survival rate of two groups **(b)** Two kaplan–meier survival curves: **(a)** overall survival curves comparing RAP (blue line) vs. 3D LAP (red line) groups over 36 months, showing comparable survival rates. **(b)** Disease-free survival curves comparing both groups, with no significant difference in recurrence rates both graphs include censored data marks (+) and confidence intervals (shaded areas).

The overall survival rate and disease-free survival for both groups are illustrated in Figures 2a,b, respectively. During follow-up of the 3D LAP group, four patients developed hepatic or pulmonary metastases, and one patient succumbed to disease 24 months post-surgery. Conversely, only one anastomotic recurrence and ten distant metastases were documented in the RAP group, with no mortality recorded throughout the follow-up period. The three-year overall survival rates were 100.0% for the RAP group and 99.1% for the 3D LAP group, with no statistically significant difference between the groups (*P* > 0.05). Similarly, the three-year disease-free survival rates were 83.2% (104/125) for the RAP group and 96.3% (105/109) for the 3D LAP group, demonstrating no statistically significant difference between the groups (*P* > 0.05).

## Discussion

Robotic-assisted surgery, representing a paradigm shift in minimally invasive surgery, has demonstrated substantial benefits in optimizing rectal cancer treatment outcomes (20, 21). Through this retrospective cohort analysis, we systematically compared the short-term efficacy of robot-assisted and laparoscopic radical rectal cancer resection. Our findings indicate that, compared with traditional laparoscopic surgery, robot-assisted surgery significantly reduced intraoperative blood loss (22). These results underscore that robotic surgery has established a new benchmark in precision and minimally invasive surgery, exhibiting superior technical advantages over traditional laparoscopic approaches (23, 24).

Despite the fundamental similarity in core surgical steps between both approaches, the time required for robotic arm setup and docking contributes to the observed prolongation of overall operative time in the RAP group (25, 26). Furthermore, the substantially elevated cost of robotic surgery compared to standard laparoscopic surgery imposes a considerable financial burden on patients and represents a significant barrier to the widespread implementation of robotic surgical systems.

Regarding postoperative pathological outcomes, no significant differences were observed between the two surgical approaches with respect to distal resection margin distances or lymph node counts. The statistically significant difference in postoperative TNM staging may be attributable to potential selection biases inherent in this single-center study. Nevertheless, robotic resection achieved radical tumor resection goals comparable to those of laparoscopic surgery.

Postoperative complications remain a critical consideration in evaluating invasive procedures (27). Analysis of complications occurring within 30 days post-surgery revealed an overall complication rate of 28.0% in the RAP group, which aligns with the 22.0% reported in a Korean study (28), supporting the consistency of our findings. The observation that the overall complication rate for robotic surgery showed no reduction compared to conventional 3D laparoscopy (20.9%) suggests that the purported advantages of robotic surgery in minimizing surgical complications warrant further investigation.

Currently, the recovery of urinary and sexual function following surgery for low rectal cancer has garnered increasing clinical attention (29–31). Our findings demonstrated that the RAP group exhibited significantly superior postoperative recovery scores at the three-month follow-up, with a clinically meaningful difference. These results suggest that although both approaches effectively preserved the anatomical integrity of pelvic autonomic nerves, the inherent limitations in visualization and instrumental maneuverability in the 3D LAP group may have resulted in inadvertent thermal or mechanical stimulation of pelvic autonomic nerves, thereby delaying functional recovery.

The superior outcomes observed in the robotic group can be attributed to several key technological advantages. First, the high-definition 3D visualization system provides enhanced magnification (up to 10–15×) and depth perception, allowing surgeons to clearly identify and preserve delicate nerve structures within the narrow pelvic cavity. Second, the EndoWrist instruments with seven degrees of freedom enable precise dissection around critical anatomical structures with minimal tissue trauma. Third, the elimination of physiological tremor and improved ergonomics contribute to reduced surgeon fatigue during prolonged procedures, potentially leading to more meticulous nerve preservation. These technical advantages collectively facilitate superior lymph node retrieval by enabling comprehensive dissection of the mesorectal tissue while maintaining optimal visualization of tissue planes.

Similarly, the IIEF score in the RAP group was significantly elevated at six months, revealing a substantial difference between the surgical groups. These findings further substantiate that the RAP group achieved superior functional recovery compared to the 3D LAP group, underscoring the advantages of the da Vinci robotic system. The robotic surgical approach likewise demonstrated accelerated recovery of female sexual function, with a statistically significant difference at the six-month follow-up. Importantly, the majority of female participants gradually regained preoperative levels of sexual function within 24 months post-surgery, establishing a valuable clinical reference for predicting the temporal pattern of female sexual function recovery following surgery.

Consistent with all oncological interventions, postoperative survival rates constitute the paramount concern. Our analysis revealed no significant difference in overall survival or disease-free survival between the two groups. These findings corroborate the results reported by Park S et al., who observed 5-year survival rates of 80.5% in the laparoscopic group and 87.6% in the robotic group, thereby confirming that long-term survival and local recurrence rates following robot-assisted total mesorectal excision were comparable (32). In conjunction with our institutional data, we provide additional evidence that both robotic and 3D laparoscopic surgical modalities can safely and effectively treat low rectal cancer, with no significant differences in short-term survival rates.

### Study limitations

This study has several limitations that should be acknowledged. First, the retrospective nature of this single-center study introduces inherent selection bias, which may have influenced patient allocation to different surgical approaches. The lack of randomization limits the strength of causal inferences that can be drawn from our findings. Second, the relatively short follow-up period (maximum 3 years) may not fully capture long-term oncological outcomes and late complications. Third, the assessment of functional outcomes relied on patient-reported questionnaires, which may be subject to recall bias and varying levels of patient comprehension. Fourth, all surgeries were performed by the same experienced surgical team, which enhances internal validity but may limit the generalizability of results to other centers with different levels of expertise. Fifth, the sample size, while adequate for detecting differences in functional outcomes, may be insufficient to detect small but clinically relevant differences in rare complications or long-term survival. Finally, the cost-effectiveness analysis was limited to direct procedural costs and did not include indirect costs such as productivity loss or quality-adjusted life years. Future prospective, multicenter randomized controlled trials with longer follow-up periods are warranted to validate these findings and provide more robust evidence for surgical decision-making in rectal cancer treatment.

## Conclusions

In conclusion, robotic surgical systems constitute a safe and feasible approach for middle and low rectal cancer treatment. They demonstrate multi-dimensional advantages including superior minimally invasive characteristics and reduced intraoperative blood loss, albeit at a substantially higher financial cost. Most significantly, robotic surgical systems enhance both short-term prognosis and functional recovery of patients through their provision of clear, magnified visualization of pelvic structures,coupled with superior instrumental dexterity that enables more precise nerve preservation and comprehensive lymph node dissection.

## Data Availability

The original contributions presented in the study are included in the article/Supplementary Material, further inquiries can be directed to the corresponding author.
